# Long-Term Management of Migraine With OnabotulinumtoxinA (Botox) vs Calcitonin Gene-Related Peptide Antibodies (Anti-CGRP)

**DOI:** 10.7759/cureus.46696

**Published:** 2023-10-08

**Authors:** Manoj R Pallapothu, Maria G Quintana Mariñez, Mohana Chakkera, Niriksha Ravi, Rajita Ramaraju, Aastha Vats, Athira R Nair, Atithi K Bandhu, Divya Koirala, Lubna Mohammed

**Affiliations:** 1 Research, California Institute of Behavioral Neurosciences & Psychology, Fairfield, USA; 2 Neurology, Pontiac General Hospital, Pontiac, USA; 3 Pediatrics, California Institute of Behavioral Neurosciences & Psychology, Fairfield, USA; 4 Internal Medicine, California Institute of Behavioral Neurosciences & Psychology, Fairfield, USA; 5 Internal Medicine and Neurology, California Institute of Behavioral Neurosciences & Psychology, Fairfield, USA; 6 General Practice, Lady Hardinge Medical College, New Delhi, IND; 7 Internal Medicine, Pontiac General Hospital, Pontiac, USA; 8 Internal Medicine, Tribhuvan University Institute of Medicine, Kathmandu, NPL

**Keywords:** calcitonin gene related peptide antibody, botox vs anti-cgrp, anti-cgrp monoclonal antibody, botox injections, migraine treatment, migraine headaches, migraine disorder

## Abstract

In this literature review, we will evaluate the effectiveness of OnabotulinumtoxinA (Botox) and anti-calcitonin gene-related peptide (anti-CGRP) in the treatment of migraine headaches. Both therapies are frequently prescribed for managing and preventing migraines and have received Food and Drug Administration (FDA) approval. The mechanism of action, side effects, compliance, cost-effectiveness, and migraine treatment provided by these two medicines were compared in the analysis of several studies. Many studies found that as Botox was administered by a doctor every three months and had fewer side effects than anti-CGRP, which is self-administered every month, it was more compliant than anti-CGRP. After examining the data, Botox is believed to be the most effective therapy. Although both therapies are efficient, this article compares them to determine which is the best management strategy.

## Introduction and background

According to several studies, it is revealed that the population suffering from migraine is 15%-17% of women and 6% of men [1]. Migraine is the second most common cause of disability worldwide and the first among young women aged 15 to 49, according to the Global Burden of Disease 2019 [2]. Migraine is a terrible neurological illness characterized by a pulsatile unilateral headache, nausea, vomiting, and sensitivity to sensory stimuli that recurs and lasts for 4-72 hours [2,3]. It is a complex, recurrent, and disabling type of neurovascular headache condition. Increased sensitivity to light, sound, and smell are all symptoms of auras, and some migraines are also linked to vomiting and nausea [2]. Several variables might cause these prodrome symptoms, including alcohol, a lack of food, perfumes, hormones, stress, and the weather [1].

The pharmacological approach to migraine can be separated between acute or prophylactic medications that aim to minimize the severity and frequency of migraine episodes [4]. Here, we will examine the two therapy alternatives: OnabotulinumtoxinA (Botox) and anti-calcitonin gene-related peptide (anti-CGRP). Botox and anti-CGRP have been approved and advised for those with persistent migraines to prevent headaches [5,6].

Around 34 years ago, the peptide calcitonin gene-related peptide (CGRP) was discovered, and it was assumed to be one of the key peptides involved in the regulation of cerebral blood vessels. A double-blind cross-over study found severe headaches in patients following the administration of human CGRP in 2002 to further identify the involvement of CGRP as a culprit behind migraine headaches. As a result, CGRP was identified as the cause of episodic migraine headaches [6]. It was finally noted that migraines were caused by CGRP, which causes inflammation and irritation of the meninges [1]. Recently, new and modified medicines known as CGRP receptor antibodies were developed [6]. Anti-CGRP has revolutionized migraine prevention. However, because of the high cost of treatment, it is necessary to keep treatment duration to a bare minimum [7].

Botox comes from *Clostridium botulinum*, which is an anaerobic, gram-positive, motile, spore-forming bacterium that, under anaerobic conditions, generates a protease exotoxin. This toxin prevents acetylcholine, a neurotransmitter involved in muscular contraction and glandular secretion, from being released from the nerve endings causing inoperative glands [8,9]. Muscle paralysis is caused by this toxin, which in turn inhibits the pain experienced with migraines. Every three months, a physician injects botulinum toxin into the pericranial muscles of patients, which makes them more compliant. It works by suppressing the release of neurotransmitters from primary-sensory neurons, thereby suppressing the pain, probably caused by excessive muscular spasms that cause recurrent migraine headaches [1]. According to the articles examined, Botox is more effective for migraine prevention. While both anti-CGRP and botox are effective and safe, botox has been studied for a longer period of time, has fewer side effects, is more cost-efficient, and is easier to use [1].

## Review

This article is a review of the literature. This section will discuss the disease pathophysiology, the OnabotulinumtoxinA (Botox) mechanism of action, the anti-calcitonin gene-related peptide (anti-CGRP) mechanism of action, and a comparison of both treatment options.

Migraine pathophysiology

The premonitory phase, which begins three days before the headache phase, is characterized by a complex interplay between numerous cortical and subcortical brain regions that control nociceptive transmission, including the hypothalamus and brainstem nuclei. Activation of the trigeminovascular system is involved in the headache phase [10]. According to the third edition of the International Classification of Headache Disorders (ICHD-3), persistent headache is defined as 15 or more days of cerebral pain per month for more than three months, with headache spikes occurring at least eight days a month [11]. The thalamus, hypothalamus, basal ganglia nuclei, and multiple brainstem levels receive signals via ascending routes from the Trigeminocervical complex (TCC). These nuclei project to a variety of cortical areas, including the somatosensory, insular, motor, parietal, retrosplenial, auditory, visual, and olfactory cortices, which are involved in processing the cognitive, emotional, and sensory-discriminative aspects of nociceptive signals and cause symptoms like extreme sensitivity to light, loud sounds, odor, touch, and cognitive dysfunction [10].

**Figure 1:**
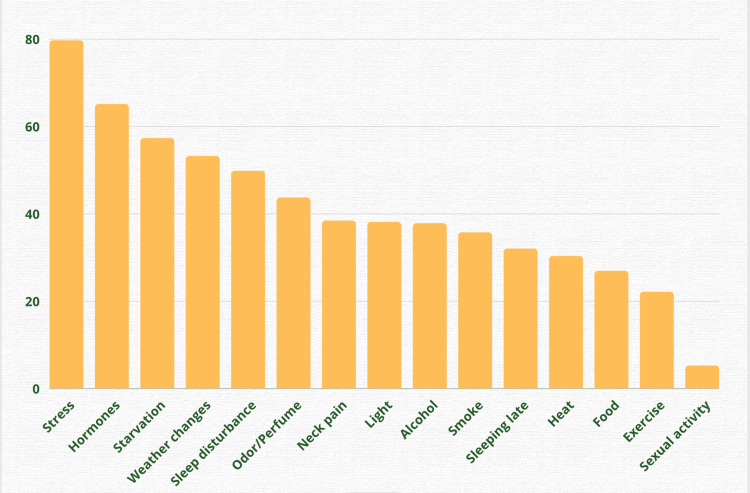
Migraine trigger analysis Figure created by author Manoj Pallapothu

The migraine triggers reported in decreasing order of frequency in one of the broadest migraine trigger analyses of 1027 migraine sufferers, whose migraine was diagnosed by the International Headache Society (IHS) criteria are given below. The strong triggers such as stress, hormonal, and a few other effects obtained in that research, are shown in the graph below with the x-axis being the triggers and the y-axis being the migraine frequency percentage (Figure 1) [12].

Non-steroidal anti-inflammatory medications (NSAIDs) are commonly used to relieve pain. A few medications, on the other hand, are used to avert migraines from happening again in the future. Many of the existing drugs administered, such as beta-blockers and antipsychotics, are off-label and not designed to treat migraines. Two new therapies that appear to be more promising in avoiding persistent migraines are CGRP monoclonal antibodies (mAbs) and botulinum toxin. In 2018, the Food and Drug Administration (FDA) authorized three CGRP mAbs to treat migraines: galcanezumab, erenumab, and fremanezumab. Botox was licensed by the FDA for migraine therapy in 2010 and has been investigated for migraine prevention for a longer time [1].

Botox mechanism of action

Botulinum toxins are exotoxins created by the *Clostridium botulinum* bacterium under anaerobic conditions [11]. A disulfide bond interfaces one heavy-chain and one light-chain polypeptide within the protein. The overwhelming chain connects to layer receptors when it comes into touch with presynaptic nerve terminals, and the toxin-receptor complex enters the cell by means of endocytosis. The disulfide association breaks and the light chain is discharged into the neuronal cytoplasm, causing the protein to change shape. After that, the light chain interatomic with the soluble N-ethylmaleimide-sensitive factor connection protein receptor (SNARE) complex is made up of a combination of vesicular and film proteins that permits synaptic vesicles to combine with the synaptic layer. Botulinum neurotoxin type-A (BoNTA) particularly cleaves synaptosomal-related protein-25 (SNAP-25), an imperative SNARE complex protein joined to the plasma membrane's cytosolic face. The toxin prevents the exocytosis of neurotransmitters and neuropeptides from nerve terminals into the synaptic cleft by attacking the SNARE complex [13]. They have also appeared to relieve automatically spasming muscles by stifling the expression of acetylcholine, the neurotransmitter that triggers muscle withdrawal at the neuromuscular intersection. They are a well-known example of neurotoxins that have a variety of FDA-approved therapeutic uses, including effective relaxation in cervical dystonia, prevention of cerebral pain, and management of spastic bladder [11].

In spite of the fact that the impacts are transitory, they may be modified by changing the recurrence and sum of the organization. Among the deadliest naturally occurring hazards, botox has caused a lot of unintentional fatalities before it was recently made available for medical use. In 1980, it was the first time it was used in medication to remedy strabismus, a disorder in which both eyes do not line up in the same direction. In spite of the fact that the aesthetic benefits of Botox on wrinkles were, to begin with, recognized in 1989, it was not until 2002 that it became worldwide conspicuousness as a conceivable restorative treatment operator taken after FDA clearance [9]. Ali et al. found that BoNTA smothers mechanical nociception in fringe trigeminovascular neurons in 2014. Trigeminal and meningeal nociceptors are thought to have a noteworthy part in the onset of headache migraines. According to Ali et al., BoNTA slows the initiation of headaches by preventing high-threshold, mechanosensitive particle channels involved in mechanical pain from intertwining into the nerve terminal layer [11].

Botox features a number of preferences over other preventative drugs. In 2018, a cohort study inspected 245 unremitting headache sufferers who were administered Botox for different cycles. Eighty patients remained within the exploration, and 82.8% of them said the botox made a difference in controlling their headaches. In 2010, a bigger randomized, double-blind inquiry with 1,384 members was conducted, with 688 patients accepting Botox and 696 receiving a placebo treatment. Patients who got Botox had a significant lessening in the recurrence of migraines. Besides, since this handle is rehashed every 12 weeks, it was found that compliance was easier. Aside from the typical botox impacts, there were no outstanding long-term antagonistic impacts detailed. Botox was found to be effective and secure in more youthful people in a trial counting youngsters. Since headaches can strike at any age, a few individuals start to endure them as early as a youth. During a clinical experiment, Botox was administered to a group of six individuals between the ages of 14 and 18 for whom previous migraine preventatives had failed. It was discovered that every sixth subject saw a reduction in headache frequency without experiencing any significant adverse effects [1].

Anti-CGRP mechanism of action

Calcitonin gene-related peptide (CGRP) is an important neurotransmitter involved in migraines. Anti-CGRP agonists and monoclonal antibodies (mAbs) can prevent migraines [1]. The nociceptive neuronal nerve ends emit CGRP, which is a vasoactive neuropeptide. Diverse isoforms of CGRP are created through hereditary locus heterogeneity. It is made by elective grafting in specific tissues and is adhered to nearby calcitonin on chromosome 11 by the calcitonin gene-related peptide Alpha (CALC I gene). Additionally, beta CGRP is delivered by the calcitonin gene-related peptide beta gene (CALC II gene), which is arranged at a distinctive area on the same chromosome. The 37 amino acid isoform of alpha CGRP is the primary target for CGRP antagonists and is only present in the central nervous system [14].

Eptinezumab, brand name Vyepti, is an anti-CGRP and is a humanized immunoglobulin (Ig)G1 monoclonal counteracting agent produced in yeast cells of Pichia pastoris utilizing recombinant DNA strategies. It has been in the advertising for around a year and is utilized to anticipate headaches. Amid a headache, the trigeminal nerve sends torment signals to the brainstem and higher-order brain regions through CGRP. As a result, it's thought that eptinezumab prevents headaches by attaching to (and hindering) CGRP particles. It can tie to both α- and β-CGRP ligands specifically and rapidly to prevent them from joining CGRP receptors; however, it is difficult to separate, which might explain its brief onset and prolonged duration of effect [2].

A clinical experiment randomly assigned 1,672 patients to different dosages of galcanezumab and a placebo group. A 120mg dosage, a 240mg dose, and a placebo were given to the patients in a 1:1:2 ratio. About 61% of participants in the 240mg group reported a 50 percent decrease in headaches [1]. It has appeared to be valuable in the anticipation and treatment of migraines [15]. In expansion, 62.3% of those given 120mg detailed the same. Gepants, a first- and second-generation CGRP receptor antagonist, produced hepatotoxicity, while the CGRP mAbs didn't seem to have any immediate short-term negative effects. A survey about the adequacy of monoclonal antibodies against CGRP found that the medicines were viable and well endured in various trials. Since monoclonal antibodies are converted into amino acids and peptides, they do not interact with other medications, making it simpler for individuals to take a number of prescription drugs. Numerous of the prior preventative drugs were utilized every day, be that as it may, these particular monoclonal antibodies against CGRP are taken once a month. This enables patients to adhere to their treatment plans [1].

Botox vs anti-CGRP

Chronic migraines are treated with calcitonin gene-related peptide (CGRP) monoclonal antibodies (mAbs) and onabotulinumtoxinA (botox). The viability, adverse effects, cost-effectiveness, and other variables of CGRP mAbs and botox will be compared in this section [1]. An add-up to 1,672 patients was arbitrarily doled out to groups with shifting dosages of galcanezumab and a placebo treatment. One-thousand six-hundred and seventy-two patients were randomly assigned into dose- and placebo groups for galcanezumab. On 240 mg, 60.9% of the patients had a >50% reduction in migraine frequency. It has been demonstrated that CGRP mAb reduces migraines without causing any negative side effects [1]. They moreover do not interact with other drugs and do not have any hepatotoxic unfavorable impacts. Additionally, it was managed once a month rather than on a regular basis, which advances compliance [1]. In a retrospective study, from 1st July 1994 to 1st December 2017, all health documentation was examined to determine whether patients with hemiplegic migraines had at least one round of botox injections. The frequency and nature of the patients' headaches before and after taking botox were noted after reviewing the clinical notes of the remaining eleven patients (four with familial hemiplegic migraine (FHM) and seven with sporadic hemiplegic migraine (SHM)). After administering botox, nine out of the 11 study patients saw a reduction in the frequency, intensity, and/or length of their auras [5]. FHM and SHM were two of the two non-responders. Six of the nine individuals who had better aura symptoms spoke about the effects of botox "wearing off" around week nine or 10 of the 12-week cycle, followed by improvements in the next cycle [5]. The comparison between Botox and anti-CGRP on admission, pain reduction, initiation, adverse effects, and compliance is shown in Table 1 [1].

**Table 1:** Comparison between Botox and anti-CGRP Botox: OnabotulinumtoxinA; Anti-CGRP: Calcitonin gene-related peptide antibodies Table created by author Manoj Pallapothu

Features	Botox	Anti-CGRP
Admission	Physician administered every 3 months	Self-administered every month
Pain reduction	✔	✔
Initiation	10-14 days	1 month
Adverse effects	Muscle weakness, ptosis, and neck pain	Constipation, rash, pruritus, and fatigue
Compliance	More	Less
Examples	—	Erenumab, Eptinezumab, Fremanezumab

In order to determine if further treatment with CGRP-mAbs enhances Botox therapy in chronic migraine sufferers, researchers in one study performed a retrospective file analysis of patients who got both Botox and a CGRP-mAb. Patients who were 18 years of age or older presented at a single headache center between May 2018 and May 2019 and met the requirements for chronic migraines according to the International Classification of Headache Disorders, third edition. Patients who received a different new medication over the course of the study or who had been receiving CGRP-mAb therapy for less than two months were excluded. The number of monthly headache days reported changed, and this was the trial's main objective. The secondary result was a change in pain intensity. In patients with chronic migraine who have only had a partial response to Botox, adjunctive preventative therapy with a CGRP-mAb drug is safe and effective. The CGRP-mAbs considerably decreased the number of headache days and pain severity, according to the researchers, with adverse event rates comparable to those seen in prior studies of these drugs [15].

It's interesting to note that many of the triggers mentioned by patients who present with migraines, such as lack of sleep, hunger, or strong light, may really be premonitory signs of an attack that is already underway. This association explicates why observations from survey-based studies, during which clients basically characterize their own perspective of factors triggering their migraine attacks, frequently differ from observations in clinical studies aimed at prospectively recognizing and vindicating migraine trigger factors [16]. The majority of current migraine prevention drugs were created to address illnesses apart from migraine. The problems with a few medicines' tolerability may be partially explained by this. Since they have demonstrated efficacy in clinical studies, first-line preventative medications from the antidepressant, antihypertensive, antiepileptic, and calcium channel blocker groups, among others, are still often administered [4]. However, none of the traditional oral preventative medications such as tricyclic antidepressants, beta blockers, serotonin (5-HT2) antagonists, ergots, and anti-epileptic drugs were developed to treat headaches, despite the fact that they provide up to 45 percent of migraineurs with a 50 percent reduction in the number of monthly days they experience headache pain. Additionally, these medications are typically not tolerated well, which results in low adherence [17].

A meta-analysis study found that the CGRP monoclonal antibody performed better than the placebo in terms of the average change from baseline in headache days (with mean deviation (WMD) = 1.94, 95 percent CI: 2.37 to 1.51, p 0.001). The difference in headache days between the botulinum toxin and placebo groups was equally statistically significant with low heterogeneity (I2 = 37%) (WMD = 1.86, 95 percent CI: 2.74 to 0.97) [18]. Even while Botox has been associated with significant clinical relief for people who suffer from chronic migraines, it typically fails to reduce headache frequency, necessitating the use of other medications [19]. The guidelines advise Botox is given in a series of between 31 and 39 microscopic injections. These are injected either under the skin or directly into the muscles of the neck, forehead, and area above the ears. Your doctor will have received training in administering Botox to treat severe migraines. Every 12 weeks, injections are administered. Botox is typically used until your migraine has become episodic for three consecutive months or when there has been a noticeable improvement in impairment as measured by quality of life surveys. If Botox doesn't sufficiently reduce your migraines, it might be stopped [20]. The same injectables that dermatologists and cosmetic surgeons use to reduce face wrinkles are also used to treat migraines. Botulinum toxin injections into various locations around the head and neck are used by qualified medical experts to treat migraines [21]. A single-use vial of powdered Botox is available. To create a liquid solution, the medication is blended. The drug is administered intramuscularly to individuals with persistent migraine headaches in order to avoid migraines and injected into various parts (shown in Figure 3 below) [22]. Drugs used as preventative measures are meant to lessen the frequency of migraine attacks [22]. According to a review of the most recent data on the role of CGRP in the digestive system, CGRP itself may lessen stomach emptying, stop rat colon contractions, and decrease food intake. Through neuronal CGRP-receptors, some of these effects were indirect, while others were local. This letter intends to further knowledge of a key gastrointestinal target for CGRP that may help to understand anti-CGRP therapy-induced constipation and may even offer a pharmaceutical option to help people with constipation feel better [23]. Therefore, most migraine experts believe that these medications affect CGRP outside of the central nervous system (CNS), maybe in the meninges (the membranes that cover the brain and skull), where migraine pain may begin [24].

**Figure 2:**
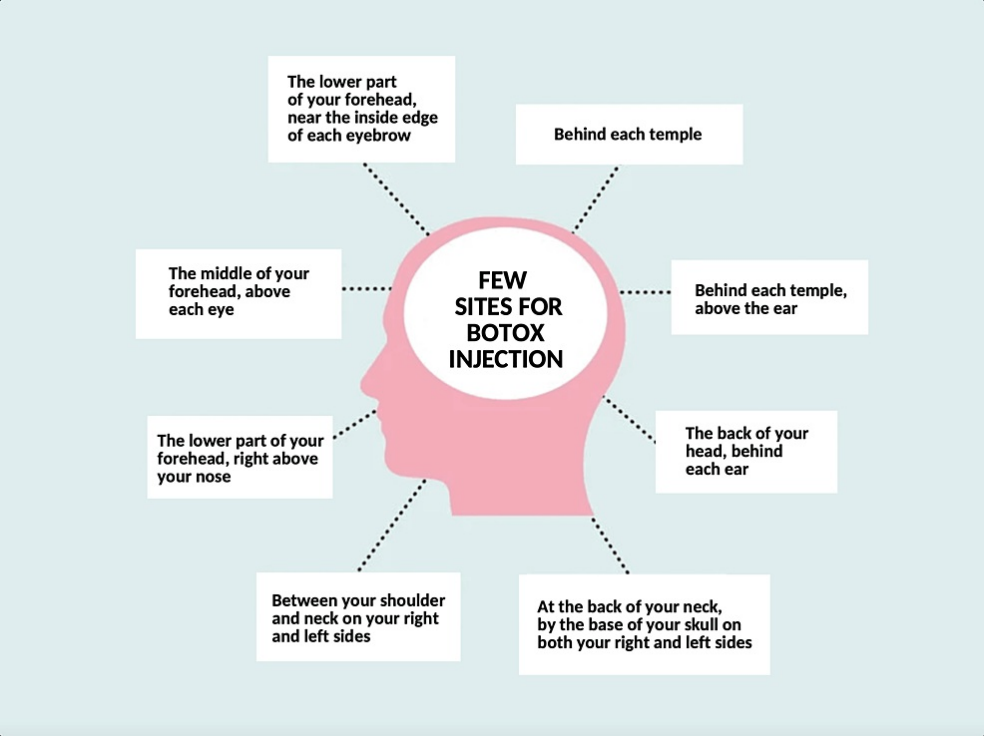
Few sites for Botox injection Botox: OnabotulinumtoxinA Figure created by author Manoj Pallapothu

Although both CGRP monoclonal antibodies and Botox are beneficial in treating migraines in distinct ways, they also have drawbacks, as indicated. Patients must self-inject CGRP mAbs once a month, and it has been proven to have certain adverse effects, including a rash and itching at the injection site. It is also a costly medicine, costing up to $600 per month without insurance, making it less accessible to some patients [1]. Your insurance provider could need proof that you've "failed" on two or three oral preventatives before granting clearance. Additionally, you might need to keep a headache diary, proving that you experience at least 15 headache days each month. Your insurer will likely want proof of improvement once the injections have begun in order to keep paying for the treatment. Depending on your plan, you might also need to go in for a follow-up appointment in between injections [25]. Because it is administered into the subcutaneous tissue, the injection is also highly unpleasant. Emgality (galcanezumab), one of the drugs with a median flow rate of 0.4mL/s over four seconds. Because a liquid is distributed into the muscle at a rapid rate, this might produce discomfort. One such random controlled trial (RCT) found that upper respiratory tract (URT) issues, exhaustion, and migraines were predominant unfavorable impacts in a considerable number including 483 people. The stoppage had a noticeable antagonistic impact on particular CGRP mAbs in more than 20% of patients in a review inquiry in 2020 [1]. Additionally, Botox has a number of drawbacks that could hinder patient compliance. In an RCT involving 1384 people, it was discovered that patients receiving botox had higher adverse effects than those receiving a placebo. Muscle wasting and neck ache are a couple of these negative effects. Plenty of the participants in this research also presented with eyelid ptosis. While this does go away eventually and is to be expected with a drug like Botox, it is still crucial to keep in mind as it might reduce patient compliance. Even if botox injections for migraines are done every three months, the total cost of therapy can be close to $3,000, making it very difficult for many individuals to afford and lowering compliance (shown in Table 2) [1].

**Table 2:** Drawbacks of Botox and Anti-CGRP Botox: OnabotulinumtoxinA; Anti-CGRP: Calcitonin gene-related peptide antibodies Table created by author Manoj Pallapothu

Botox	Anti-CGRP
Muscle weakness	Fatigue and constipation
Neck pain	URT symptoms
Eyelid ptosis	Rash and pruritus at the injection site
Costs $3000 every 3 months	Costs up to $600 every month
Effectiveness affected by high BMI	Painful as injected subcutaneously

Whereas these two therapeutic alternatives work in particular ways, they appear to have a comparable influence on blocking the nociceptive pathways involved in cerebral pains. Whereas CGRP mAbs are theorized to stifle the sense of torment by irritating a noticeable fiery marker such as CGRP, botox accomplishes so by means of diminishing the discharge of acetylcholine in neuromuscular intersections. Later trials that explored the adequacy of CGRP mAbs and had a huge test measure demonstrated that these medicines were solid preventatives for diligent headaches. Botox has been illustrated to be useful in numerous inquiries over the past decade. With respect to side impacts, both CGRP mAbs and botox have direct short-term antagonistic impacts; all things considered, one long-term negative impact distinguished in people who utilized CGRP mAbs was a stoppage. In comparison to botox infusions, the CGRP mAb infusions are moreover altogether more excruciating. Cost-effectiveness ought to be taken into consideration when contrasting these two drugs. Those without insurance might not be able to buy either medication, indeed in spite of the fact that a few insurances mostly or totally cover both medicines. For individuals with restricted budgets, Botox may be a practical elective since it is by and large the less costly medicine over the long pull. Agreeing with the publications inspected, botox may be a superior treatment choice for migraines than CGRP mAbs since it is more effective, less costly, and effortless [1].

Limitations

Although we performed a thorough bibliometric analysis using the platform available, we cannot completely rule out the chance that we missed important research because we only included articles from a small number of databases. The research covered in our study was all in the English language. The comparison of botox with anti-CGRP was covered in a small number of research papers. To properly compare these two, we should strive to do a more in-depth study.

## Conclusions

Migraine is a devastating condition that many people across the universe suffer from. The two major therapies used in the current management system are OnabotulinumtoxinA (Botox) and anti-calcitonin gene-related peptide (Anti-CGRP). These two options can be used for long-term management or for preventive use. Botox is an exotoxin, produced by *Clostridium botulinum*, that blocks the acetylcholine release from the nerve endings, thus making the glands inoperable. Whereas, anti-CGRP works by inhibiting the inflammatory receptor, thereby inhibiting the pain sensation. Both Botox and anti-CGRP have few side effects. The main side effect of anti-CGRP is constipation and upper respiratory tract infections with other noticeable side effects like rash pruritus and fatigue. Botox also has side effects like ptosis, neck pain, and muscular weakness. Anti-CGRP being administered every month by the patient led to less compliance when compared to Botox, which was physician-administered every three months. Anti-CGRP also caused pain at the injection site, making patients wantingly skip the dosage, if the migraine symptoms were lighter. Botox and anti-CGRP also have an overall cost difference, with Botox being cost friendly in a long run. Insurances may or may not cover the treatment, but overall botox is better when compared. Anti-CGRP can be given along with Botox, which was proved to be better as it had fewer side effects, and also patients showed better results. Finally, Botox is a step better than anti-CGRP but both can be used together too for a better result.
